# No association between food allergens in the complementary feeding diet and eczema during the first 12-months in the Cork BASELINE Birth Cohort

**DOI:** 10.1186/2045-7022-5-S3-O18

**Published:** 2015-03-30

**Authors:** Sinéad M O’Donovan, Deirdre M Murray, Jonathan O’B Hourihane, Louise C Kenny, Alan D Irvine, Mairead Kiely

**Affiliations:** 1 Vitamin D Research Group, School of Food and Nutritional Science, University College Cork, Ireland; 2 Department of Paediatrics and Child Health, University College Cork, Ireland; 3 The Irish Centre for Fetal and Neonatal Translational Research; 4 Department of Obstetrics and Gynaecology, University College Cork, Ireland; 5 Department of Clinical Medicine, Trinity College, Dublin, Ireland; 6 Department of Paediatric Dermatology, Our Lady's Children's Hospital, Dublin, Ireland; 7 National Children’s Research Centre, Dublin, Ireland

## Objective

To describe the allergenic food ingredients present in complementary feeding and to examine their potential association with persistent eczema.

## Method

Prospective data on complementary feeding practices, using a detailed food diary over the first 6-weeks of complementary feeding, were collected as part of Cork BASELINE Birth Cohort Study (*n* 1537). An allergenic food was determined present, if labelled as an ingredient and not if stated as ‘may contain’. Eczema was determined if the UK working party diagnostic criteria were satisfied at 6 and 12 months.

## Results

Data are described for the 823 infants for whom a food diary was completed. The point prevalence of eczema at 6 and 12 months was 16 and 14%, respectively. 8.3% had persistent eczema at 12 months. Most infants (79%) were introduced to solids between 17-26 weeks, 18% <17 weeks. At least one allergenic food ingredient was provided to 64% of infants; including cow’s milk (56%), wheat (43%), soy (41%), eggs (10%), fish (8%) and kiwi (7%). Infant breakfast cereals were the main source of exposure to cow’s milk (71%), wheat (92%) and soy (98%). First exposure to an allergen was typically between 17-26 weeks (73%) and 26% were exposed after 26 weeks. Mothers <25 years (84 vs 70%), who were smokers (83 vs 69%) and had no university degree (82 vs 64%) were more likely to give an allergenic food ingredient in the complementary feeding diet (all P<0.05). There was no association between exposure to allergenic food ingredients and persistent eczema [OR 0.96 (0.57, 1.63)] or between exposure either before 17 weeks [OR 2.43 (0.58, 21.5)] or after 26 weeks [OR 1.29 (0.67, 2.5)] and persistent eczema, compared with 17 to 26 weeks (all P>0.05).

## Conclusions

Infant breakfast cereals were the main source of cow’s milk, wheat and soy during the first 6 weeks of transition to solid food. Exposure to allergenic food ingredients in the initial stage of complementary feeding had no influence on persistent eczema. Clear guidance on the introduction of allergenic food ingredients in foods labeled as appropriate from 4-6 months is required.

